# Failure modes of patellofemoral arthroplasty—registries vs. clinical studies: a systematic review

**DOI:** 10.1080/17453674.2019.1634865

**Published:** 2019-07-01

**Authors:** Nikolaj B Bendixen, Peter W Eskelund, Anders Odgaard

**Affiliations:** Department of Orthopedic Surgery, Copenhagen University Hospital Herlev-Gentofte, Hellerup, Denmark

## Abstract

Background and purpose — Patellofemoral arthroplasty (PFA) has been debated since early studies showed poor implant survival. Recent studies show better results. This review reports failure modes for PFA and investigates differences in data reported from registries and clinical studies. Additionally, we report differences in failure modes among implant designs.

Methods — A systematic search was performed in September 2018. All studies and registers describing failure modes of PFA were included and implant design was noted for each revision.

Results — This review includes 1,299 revisions of a primary PFA reported in 47 clinical studies and 3 registers. The failure modes were: 42% OA progression, 16% pain, 13% aseptic loosening, 12% surgical error, 4% wear, 2% infection, 2% broken patellar component, 1% stiffness, 1% fracture, and 7% other. The data from registries and cohort studies differed statistically significantly in 7 out of 12 failure modes. Significant differences were found in several failure modes among implant designs.

Interpretation — OA progression is the most common failure mode of PFA. There are significant differences in data on failure modes between registers and protocolled studies, notably for surgical error. The implant design significantly influences several of the failure modes. In conclusion, indication, surgical technique, and implant design are important for a successful PFA, and register-based failure modes should be interpreted with caution.

Isolated patellofemoral osteoarthritis (PF-OA) predominantly affects women, the elderly, and young adults with dysplasia or former trauma. 11% of male patients and 24% of female patients with symptomatic knees have PF-OA (McAlindon et al. 1992) and could therefore be candidates for patellofemoral arthroplasty (PFA).

Controversy concerning PFA has led surgeons to choose total knee arthroplasty (TKA) in patients with PF-OA. Recent studies on PFA show good results, however, with high survival rates and satisfying patient-reported outcomes (Goh et al. 2015, Halai et al. 2016, Konan and Haddad 2016, Osarumwense et al. 2017, Odgaard et al. 2018), suggesting that PFA may be a good choice in suitably selected patients. A rise in PFA operations has been seen in Denmark from 0.6% of primary arthroplasties in 2012, to 1.3% in 2016, but the reported prevalence of PF-OA (McAlindon et al. 1992) suggests that PFA is under-used. Register studies show poor survival of PFA compared with TKA where the 2-year survival is 91% and 97% and 10-year survival is 73% and 93%, respectively (Odgaard et al. 2017).

Because of this controversy, we performed a systemic review with the following purposes: (1) to investigate the reasons for revisions of PFA, (2) to highlight possible differences in data from registries and protocolled studies, and (3) describe differences in failure modes among implant designs.

Van der List et al. (2017) published a similar review. In our review we present newer results, a higher number of revisions (1,299 vs. 938), and, as van der List et al. suggested, we investigate the impact of implant design on failure modes. Furthermore, we focus on the differences between data from registries and protocolled studies.

## Methods

### Search strategy

A systematic search was performed in September 2018. Electronic databases (PubMed, Embase, and Cochrane Library) were searched with the terms “arthroplasty AND (patellofemoral OR PF OR PFA OR PFR) AND (outcome OR functional outcome OR scores OR results OR revision OR revision rate OR reoperation OR treatment failure OR prosthesis failure OR failure OR failure rate OR survivorship OR survival).”

16 additional articles included in the protocol for the RCT study of Odgaard et al. (2018) were used for a cited reference search (Arciero and Toomey 1988, Grelsamer 1990, Argenson et al. 1995, Krajca-Radcliffe and Coker 1996, Tauro et al. 2001, De Winter et al. 2001, Smith et al. 2002, Kooijman et al. 2003, Merchant 2004, Board et al. 2004, Ackroyd and Chir 2005, Argenson et al. 2005, Blazina et al. 2005, Cartier et al. 2005, Ackroyd et al. 2007, Merchant et al. 2017). Articles were selected by relevance and had to be registered on Web of Science.

NBB and PWE scanned titles and abstracts for relevance, and full texts were evaluated against the eligibility criteria. Included articles were checked for eligibility by AO. Studies found during the search were screened for any further studies containing relevant data. Final consensus on inclusion was reached between the authors.

Finally, registries from Scandinavia, England/Wales, The Netherlands, New Zealand and Australia were screened for information, last accessed on December 17, 2018.

The review was conducted in accordance with PRISMA guidelines and is registered on PROSPERO (CRD42018115774).

### Inclusion criteria

All studies reporting reasons for revision of PFA were included. A revision was defined as surgery where single or multiple components of the implant were removed, replaced, or supplemented. Studies in languages other than English were included if data was extractable by the authors.

### Exclusion criteria

Studies using the same cohort or database as in an already included study were excluded to eliminate the risk of patients being counted repeatedly.

### Data collection

Parameters included: authors, year published, year cohort started/ended, mean years of follow-up, number of arthroplasties, number of revisions, type of implant, and reasons for revision. 26 different failure modes were identified. These were categorized into 12 groups (Table 1, see Supplementary data).

**Figure:**
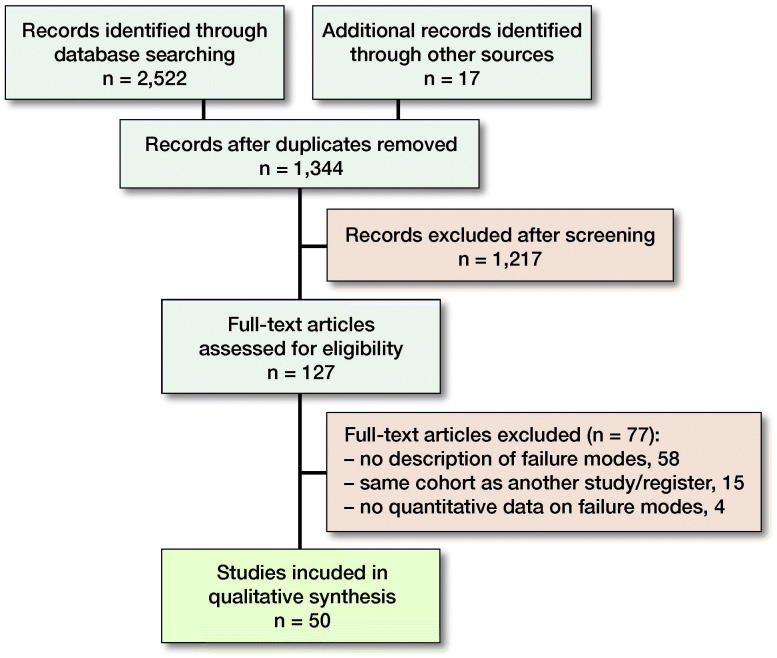
Flowchart.

### Quality of studies

All studies were assessed for quality by NBB and PWE according to Grading quality of evidence and strength of recommendations (GRADE).

### Statistics

A pooled analysis was performed for each failure mode. Final groups of failure modes were presented in percentages. A chi-square test was performed in accordance to outcomes: (1) Failure modes in cohorts vs. registries and (2) Failure modes between each implant design vs. all others. In samples where expected frequencies were lower than five, Fisher’s exact test was used. P-values < 0.05 were considered significant. All statistical analyses were performed in Excel 2016 (Microsoft Corp., Redmond, WA, USA).

### Funding and potential conflicts of interest

Institutional support was received from Stryker European Operations BV for studies on patellofemoral implants. Stryker had no influence on data accumulation, analysis or interpretation.

## Results (Tables 2–8, see Supplementary data)

### Search results

The primary search identified 2,522 studies and 17 additional studies were located by the cited reference search and by screening of references. 1,344 unique articles were found, and 1,217 articles were excluded during the screening of titles and abstracts. 127 articles were assessed for eligibility, and 50 articles (3 registers and 47 clinical studies) were included (Figure). Only the New Zealand, Australian, and English registry had relevant data according to the inclusion criteria (Table 2) (Muir et al. 1999, Baker et al. 2012, Australian Orthopaedic Association 2017)

### Quality of studies

The search revealed 1 level I RCT study, 31 level II prospective cohort studies, 14 level III–IV observational studies, and 1 level VI case study (Table 3). The studies were published between 1996 and 2018. Heterogeneity existed in the follow-up periods and types of implants used. The quality of evidence was, according to GRADE guidelines, low.

#### Failure modes of the patellofemoral implant

47 clinical studies and 3 registers containing 1,299 revisions of PFAs were found. The most common failure modes were OA progression (42%), pain (16%), aseptic loosening (13%), and surgical error (12%). Failure modes varied among registries (Table 2).

### Registries vs. clinical studies

Statistically significant differences were found between registers and clinical studies in 7 out of 12 failure modes (Table 4). Pain, aseptic loosening, operational flaws, and broken patellar component were all found to be highly significant (p < 0.001). Additionally, significant differences were observed with progression of OA and infection (p = 0.009 and p = 0.04, respectively).

Pain and aseptic loosening were more often reported as indication for revision in registers (20% vs. 10% and 17% vs. 7%, respectively). In clinical studies, progression of OA and surgical errors were more dominant than in registers (48% vs. 38% and 21% vs. 7%, respectively) (Table 4).

### Failure modes and implant design

40 studies reported data on specific implants (Table 5). 7 studies did not relate cause for revision and implant design and were excluded in this outcome. 64 revisions were excluded from clinical studies, and all 807 revisions from registers were excluded in the same manner. 428 revisions were included (Table 5) and chi-square and Fisher’s tests were performed on these. 5 implant designs (Avon, Richards, Lubinus, Autocentric, and LCS) had more than 30 documented revisions, and these were chosen for further analysis (Tables 5 and 6).

The Avon implant was revised more often (p = 0.02) for OA progression with relative risk (RR) = 1.4, and less for surgical errors (p = 0.001) with RR = 0.2. The Richards design was, with RR = 0.8, revised less for pain (p = 0.007). The Lubinus design was more often revised because of surgical errors (p < 0.001) with RR = 3.9 and wear (p = 0.001), RR = 4.3, and less often for OA progression (p = 0.005) RR = 0.5, pain (p = 0.03) RR = 0.2, and none for aseptic loosening (p = 0.008). The Autocentric implant had a higher incidence of aseptic loosening (p < 0.001), RR = 4.9, infection (p = 0.007), RR = 14 and was the only design with stiffness as a failure mode (p < 0.001), while 0 was revised because of pain (p = 0.04). The LCS implant had a greater revision rate for pain (p < 0.001) RR = 5.5 and surgical errors (p = 0.002), while progression of OA (p < 0.001) was seen less frequently with RR = 0.2.

## Discussion

### Progression of osteoarthritis as the main failure mode

This review found the most frequently reported reason for revision of PFA to be OA progression with a proportion of 42% (48% in studies and 38% in registries). Because some revised knees in this group may have been reported with the nonspecific failure mode of pain, this could even be an underestimation. Progression of OA can be viewed in 2 ways: (1) as a victory for the implant in long-term follow-up studies since it survived implant-dependent failure modes, and (2) as poor patient selection in studies with short-term follow-up.

When deciding between PFA and TKA, strict patient selection is suggested (Mohammed et al. 2008, Goh et al. 2015, Odgaard et al. 2017). Patients having a PFA for primary OA may have a higher incidence of progression of TF-OA compared with patients having surgery for dysplasia or previous fracture (Smith et al. 2002, Argenson et al. 2005). Progression of TF-OA has been noted mainly in patients with malalignment compared with a neutral hip–knee–ankle angle (Cartier et al. 2005). Studies including patients with TF-OA (Leadbetter et al. 2009, Williams et al. 2013, Dahm et al. 2014) reported a higher incidence (75%) of patients revised because of OA progression. These findings support the importance of strict patient selection. To identify patients with isolated PF-OA, Cartier et al. (2005) claimed that medial knee pain ascending stairs always indicates PF-OA, and pain descending stairs is of TF-OA origin. Bone-on-bone on tangential (skyline) radiographs is the deciding radiographic proof of PF-OA (Odgaard et al. 2017, Cuthbert et al. 2018). We encourage thorough clinical and imaging investigations preoperatively, and stress radiography, specialized radiographic projections, and MRI should be considered to ensure the absence of TF-OA.

Odgaard et al. (2018) published early results of a double-blinded RCT comparing PFA and TKA for isolated PF-OA. They concluded that patients treated with PFA obtained better knee function, better satisfaction, and larger knee-related quality of life compared with TKA within the first 2 years. The study found similar short-term survival rates for the 2 implants. Cartier et al. (2005) reported no complications in getting back to daily activities and sports after PFA surgery. Additionally, studies show that revision of PFA to TKA does not present any particular difficulties (van Jonbergen et al. 2009, Parratte et al. 2015). Therefore, it could be argued that PFA should have a place in the treatment of knee OA as a temporary implant towards TKA (Argenson et al. 2005, Leadbetter et al. 2009). Using the implant as a stepping stone could give the patient some years with a higher knee-related quality of life without compromising the results of a future TKA. However, a recent study on a cohort from the Australian registry shows that the risk of re-revision of a secondary TKA from a PFA is larger than the risk of a revision of a primary TKA (Parker et al. 2019), but the extent of confounding in register studies is unknown. Only longitudinal protocolled studies with time-weighted outcomes, e.g., area under the curve for PRO data, can determine the better strategy.

In line with results published by van der List et al. (2017) we found that progression of OA is the key failure mode for PFA. Our data, however, show a lower rate of OA progression (42% vs. 49%) and a higher rate of pain (16% vs. 6%), which indicates that the 2 populations differ. This review consists of newer data and a larger population.

### Registries vs. clinical studies

In 7 of 12 failure modes, we found a statistically significant difference between register and cohort studies (Table 4). The difference may be explained by the way indications for revisions are recorded. Where registries use fixed modes of failure, studies tend to record more specific indications. Register data (Muir et al. 1999, Baker et al. 2012, Australian Orthopaedic Association 2017) are almost devoid of surgical errors and have a high rate of pain as indication, suggesting that surgeons have a preference for reporting the nonspecific symptom rather than surgical errors. It may reasonably be assumed that surgeons have a higher tendency to scrutinize indications for patients included in a clinical study. The variation in failure modes among registers suggests that registration practice or indications vary. Cohort studies (e.g. van Jonbergen et al. 2010) investigate a specific implant, its survival, and its failure modes. They report a high rate of OA progression, surgical error, and wear. No patients were revised by the nonspecific indication pain, suggesting that focus on diagnostics could be different in cohort studies vs. the daily registration for registers.

Van der List et al. (2017) reported OA progression as the most common failure mode with more than 5 years’ follow-up. Most clinical studies included in our review have a short follow-up, and therefore include a large proportion of early revisions. The included registries date back to 1999–2003 and have no defined end follow-up. Therefore, it would be expected that registries would contain a larger proportion of revisions with more than 5 years’ follow-up and therefore a higher proportion of OA progression. The conflict between this expectation and actual register data supports the argument that revisions are reported with the less specific indication of pain.

The year of operation could influence the cause for revision. Our cohorts report data back to 1972 (Argenson et al. 2005), and the earliest registries start in 1999 (Muir et al. 1999, Australian Orthopaedic Association 2017). The high rate of surgical errors in the studies could be caused by early, less successful implant designs. An example is the Lubinus implant where Tauro et al. (2001), in a cohort from 1989–1995, reported a 71% failure by maltracking and called the implant “Unforgiving.” They even converted the trochlear component later in their study, using the left component on the right knee and vice versa.

The review by van der List et al. (2017) reports the same incoherence between registries and studies. The only difference in data is infection, which in our review is more often reported from registries.

### Failure modes and implant design

OA progression may be a success criterion for the implant design or suggest poor patient selection. A high percentage of revisions for OA progression in cohort studies with a long follow-up period (> 10 years) suggests a satisfactory design. This was observed in the study by Kooijman et al. (2003), where the Richards II design has a 75% failure by OA progression, and in the cohort from Nicol et al. (2006) who reported an 80% failure by this mode with the Avon design.

With a 56% rate of surgical error as failure mode (Table 6), the Lubinus design has met with controversies. More authors have questioned the anatomical design of the femoral component and agree that a non-anatomical design such as Avon or Richards is preferable (Tauro et al. 2001, Board et al. 2004, Cartier et al. 2005). Tauro et al. argue that the anatomical and asymmetrical design of the femoral groove in the component impairs the surgeon’s ability to correct an existing anatomical dysplasia with maltracking.

The results of LCS and Autocentric (Table 6) may indicate constructional flaws. The mobile bearing LCS design has a metal-backed patellar component (Charalambous et al. 2011). Yadav et al. (2012) argue that rotational freedom of the polyethylene could lead to locking in the trochlear groove and may cause mechanical problems and wear. Charalambous et al. (2011) found overgrowth of soft tissue in several of their revisions, resulting in a blocking of the rotation of the polyethylene, which may have caused the high frequency of pain in their study. Furthermore, extensive metallosis was found in 3 of 17 revised knees, likely caused by the metal-backed patella. Because of non-satisfactory results, Australia stopped using the LCS implant in 2009 (Australian Orthopaedic Association 2017). In studies concerning the Autocentric implant, aseptic loosening was the dominant failure mode (Table 6) (De Cloedt et al. 1999, Argenson et al. 2005), which may give the impression of design problems.

### Limitations and bias

This study has several limitations, mainly the problem of inclusion. We strove to include as many revisions as possible, even a revision from a biased case study reporting only a single failure mode. On these grounds we cannot exclude confounders and bias in general. To ensure that data of a cohort are only used once, we had to exclude some studies knowing that we lost data. It was not possible to detect duplicates between registries and studies, so this is also a bias in our study.

This review’s first outcome has similarity to the article published by van der List et al. (2017), but some inconsistencies were found with inclusion of patients and extraction of data in their paper. An example is data from Kooijman et al. (2003) where data on 19 revisions were extracted, but only 10 of these fulfilled their definition of revision, which was conversion to a TKA.

### Conclusion

Progression of TF-OA is the most common failure mode of PFA. The data from registries and cohort studies differ significantly in several modes of failure, notably for surgical error. The implant design has a significant influence on several of the failure modes. In conclusion, indication, surgical technique, and implant design are important for a successful PFA.

### Supplementary data

Tables 1–8 are available as supplementary data in the online version of this article, http://dx.doi.org/10.1080/17453674.2019.1634865

The article and search were made by NBB and PWE and were supervised and edited by AO.

*Acta* thanks Kirill Gromov and Frank Madsen for help with peer review of this study

## Supplementary Material

Supplemental Material
