# Supplementary material for: Failure modes of patellofemoral arthroplasty—registries vs. clinical studies: a systematic review
Source: Acta Orthop. 2019 Jul 1;90(5):473–8. doi: 10.1080/17453674.2019.1634865 (PMC6746256; doi:10.1080/17453674.2019.1634865)
Supplement: Supplemental Material [file IORT_A_1634865_SM1970.pdf]

Supplementary data

Table 1. Categorization of failure modes

OA progression
Pain
Aseptic loosening: lysis
Surgical error: malpositioning, mechanical,"clunking", dislocation, too small patellar button, maltracking, subluxation, instability, dissociation
Wear
Infection
Stiffness
Fracture: trauma
Rheumatoid arthritis
Broken trochlear component
Broken patellar component
Other: lateral femur necrosis

Table 3. Quality of studies: GRADE

Grade	Studies
I	Odgaard et al. 2018
II	Krajca-Radcliffe and Coker 1996, Mertl et al. 1997, De Cloedt et al. 1999, De Winter et al. 2001, Smith et al. 2002, Kooijman et al. 2003, Board et al. 2004, Argenson et al. 2005, Cartier et al. 2005, Lonner et al. 2006, Jørgensen et al. 2007, Gadeyne et al. 2008, Mohammed et al. 2008, Leadbetter et al. 2009, Butler and Shannon 2009, Sarda et al. 2011, Charalambous et al. 2011, Hutt et al. 2012, Mont et al. 2012, Hofmann et al. 2013, Morris et al. 2013, Williams et al. 2013, Davies 2013, Philippe and Caton 2014, Dahm et al. 2014, Goh et al. 2015, Hoogervorst et al. 2015, Willekens et al. 2015, Osarumwense et al. 2017, Christ et al. 2017, Metcalfe et al. 2018
III–IV	Tauro et al. 2001, Nicol et al. 2006, Hendrix et al. 2008, Starks et al. 2009, van Jonbergen et al. 2009, Odumenya et al. 2010, Yadav et al. 2012, Al-Hadithy et al. 2014, Benazzo et al. 2014, Akhbari et al. 2015, Ahearn et al. 2016, Halai et al. 2016, Konan and Haddad 2016, Middleton et al. 2018
VI	Arumilli et al. 2010

Table 2. Failure modes in total

Failure modes	Studies total	Registries total	England/Wales 2012	Australia 2017	New Zealand 2017	Total, n (%)
OA progression	235	307	22	285	0	542 (41.7)
Pain	51	158	72	73	13	209 (16.1)
Aseptic loosening	35	137	26	108	3	172 (13.2)
Surgical error	10	153	8	15	0	154 (11.8)
Wear	21	31	12	19	0	52 (4.0)
Infection	6	24	6	16	2	30 (2.3)
Stiffness	4	7	7	0	0	11 (0.8)
Fracture	6	6	6	0	0	12 (0.9)
Rheumatoid arthritis	2	0	0	0	0	2 (0.2)
Broken trochlear component	1	0	0	0	0	1 (0.1)
Broken patellar component	1	23	0	23	0	24 (1.8)
Other	29	61	5	56	0	90 (6.9)
Total	492	807	164	595	18	1,299 (100)

Table 4. Failure modes in registries vs. clinical studies (p-values from chi-squared test)

Failure modes (%)	Studies n = 492	Registries n = 807	p-value
OA progression	40,7	38.0	0.009
Pain	10.4	19.6	< 0.001
Aseptic loosening	7.1	17.0	< 0.001
Surgical error	20.5	6.6	< 0.001
Wear	4.3	3.8	0.7
Infection	1.2	3.0	0.04
Stiffness	0.8	0.9	0.9
Fracture	1.2	0.7	0.4
Rheumatoid arthritis	0.4	0.0	0.07
Broken trochlear component	0.2	0.0	0.2
Broken patellar component	0.2	2.8	< 0.001
Other	5.9	7.6	0.3

Table 5. Failure mode for each implant

Failure modes	Avon [a]	Richards [b]	Lubinus [c]	Autocentric [d]	LCS [e]	FPV [f]	Sigma [g]	Natural Knee [h]	Hermes–Ceravor [i]	Other [j]	Total
OA progression	97	51	16	17	3	8	2	0	3	10	207
Pain	17	1	1	0	12	6	1	0	0	1	39
Aseptic loosening	14	6	0	11	2	0	0	0	0	0	33
Surgical error	9	20	35	5	14	0	1	0	0	3	87
Wear	6	5	9	0	0	0	0	1	0	0	21
Infection	0	1	0	3	0	0	0	0	0	1	5
Stiffness	0	0	0	4	0	0	0	0	0	0	4
Fracture	2	0	1	0	0	1	0	2	0	0	6
Rheumatoid arthritis	0	0	0	0	0	2	0	0	0	0	2
Broken trochlear component	0	0	0	0	0	0	0	0	0	1	1
Broken patellar component	0	0	0	0	1	0	0	0	0	0	1
Other	21	0	1	0	0	0	0	0	0	0	22
Total	166	84	63	40	32	17	4	3	3	16	428

[a] Stryker Orthopedics, Mahwah, NJ, USA (Lonner et al. 2006, Nicol et al. 2006, Starks et al. 2009, Leadbetter et al. 2009, Odumenya et al. 2010, Sarda et al. 2011, Mont et al. 2012, Dahm et al. 2014, Akhbari et al. 2015, Willekens et al. 2015, Konan and Haddad 2016, Metcalfe et al. 2018, Middleton et al. 2018, Odgaard et al. 2018)
[b] Smith & Nephew, Memphis, TN, USA (De Winter et al. 2001, Kooijman et al. 2003, Cartier et al. 2005, Jørgensen et al. 2007, van Jonbergen et al. 2010, Hoogervorst et al. 2015)
[c] Waldemar Link, Hamburg, Germany (Tauro et al. 2001, Smith et al. 2002, Board et al. 2004, Lonner et al. 2006, Hendrix et al. 2008
[d] Medinov, Roanne, France (De Cloedt et al. 1999, Argenson et al. 2005, Lonner et al. 2006)
[e] (Low Contact Stress)—Depuy, Warsaw, IN, USA (Lonner et al. 2006, Arumilli et al. 2010, Charalambous et al. 2011, Yadav et al. 2012)
[f] (Femoro-Patella Vialla)—Wright Medical Technology, Arlington, TN, USA (Davies 2013, Williams et al. 2013, Al-Hadithy et al. 2014)
[g] Depuy, Warsaw, IN, USA (Goh et al. 2015)
[h] Zimmer, Warsaw, IN, USA (Hofmann et al. 2013)
[i] Roissy-en-France, France (Philippe and Caton 2014)
[j] (Krajca-Radcliffe and Coker 1996, Butler and Shannon 2009, Ahearn et al. 2016, Osarumwense et al. 2017)

Table 6. P-values and percentages for each implant

	Avon [a] 1996		Richards [b] 1976		Lubinus [c] 1975		Autocentric [d] 1977		LCS [e] 1997	
Failure modes	(%)	p-value	(%)	p-value	(%)	p-value	(%)	p-value	(%)	p-value
OA progression	58	0.02	61	0.07	25	0.005	43	0.6	9	< 0.001
Pain	10	0.5	1	0.007	2	0.03	0	0.04	38	< 0.001
Aseptic loosening	8	0.7	7	0.8	0	0.008	28	< 0.001	6	0.8
Surgical error	5	< 0.001	24	0.4	56	< 0.001	13	0.2	44	0.002
Wear	4	0.3	6	0.6	14	0.001	0	0.1	0	0.2
Infection	0	0.08	1	1.0	0	0.4	8	0.007	0	0.5
Stiffness	0	0.1	0	0.3	0	0.4	10	< 0.001	0	0.6

[a–e] See Table 5

Table 7. Total number of implants and revisions for each design

Implant design	Implants in total	Revisions
Avon [a]	1,273	161
Richards [b]	395	87
Lubinus [c]	115	32
LCS [e]	105	30
Autocentric [d]	102	37

[a–e] See Table 5

Table 8. Studies, published, period, and follow-up. Data in the table exclusively include data from clinical studies where implant design, total surgeries, and total revisions were extractable. The different cohorts included in this study do not have the same length of follow-up, and cannot be used for further analysis of survival rate etc.

Author	Period	Follow-up (years)	Implant design
**Studies**			
Krajca-Radcliffe and Coker 1996	1975–1991		Bechtol I & II
Mertl et al. 1997	1988–1994	3	Unknown
De Cloedt et al. 1999	1986–1995		Autocentric
De Winter et al. 2001	1978–1997	11 (1–20)	Richards II
Tauro et al. 2001	1989–1995	7.5 (5–10)	Lubinus
Smith et al. 2002	1992–1998	4.1 (0.5–7.5)	Lubinus
Kooijman et al. 2003	1977–1983	17 (15–21)	Richards II
Board et al. 2004	1994–1999	1.6 (0.2–4.7)	Lubinus
Argenson et al. 2005	1972–1990	16.2 (12–20)	Autocentric
Cartier et al. 2005	1975–1991	10 (6–16)	Richards II & III
Lonner et al. 2006	1997–2003	3.1 (2–5.2)	Multiple
Nicol et al. 2006	1996–1999	7.1 5.5–8.8)	Avon
Jørgensen et al. 2007	1996–1999		Richards I & II
Gadeyne et al. 2008	1986–2007		Unknown
Hendrix et al. 2008	1996–2002	5 (3–7.3)	Lubinus
Mohammed et al. 2008	1997–2006	4 (0.5–8)	Unknown
Butler and Shannon 2009	1994–2002	5	Custom-Fit
Leadbetter et al. 2009	2001–2007	3 (2–6)	Avon
Starks et al. 2009	2002–2007	2	Avon
Arumilli et al. 2010			LCS
Odumenya et al. 2010	1998–2007	5.3 (2–10)	Avon
van Jonbergen et al. 2010	1976–2005	13.3 (2–30.6)	Richards II
Charalambous et al. 2011	2004–2008	2.1 (0.4–5)	LCS
Sarda et al. 2011	2002–2007	4.5 (3–8)	Avon
Hutt et al. 2012			Unknown
Mont et al. 2012	2001–2006	7 (4–8)	Avon
Davies 2013	2007–2010	2–5	FPV
Hofmann et al. 2013	2010–2011	2.5	Natural Knee II
Morris et al. 2013	2004–2008	4 (2–6)	Avon
Williams et al. 2013	2007–2011	2.1 (0.5–4)	FPV
Al-Hadithy et al. 2014	2006–2012	3	FPV
Benazzo et al. 2014	2007–2012	4.7 (2–6)	Unknown
Dahm et al. 2014	2004–2008	4 (2–6)	Avon
Philippe and Caton 2014	1997–2003	12 (10–16)	Hermes
Akhbari et al. 2015	2001–2011	5.1 (1–10.3)	Avon
Goh et al. 2015	2008–2012	4.1 (2.2–6.1)	Sigma
Hoogervorst et al. 2015	1998–2007	9.7 (2.2–18.8)	Richards II
Willekens et al. 2015	2004–2010	4.6 (1.9–8.8)	Avon
Ahearn et al. 2016	2005–2009	7 (5–8.8)	Journey
Halai et al. 2016	2007–2011	3.2	FPV
Konan and Haddad 2016		7.1 (5–11)	Avon
Christ et al. 2017			Unknown
Osarumwense et al. 2017	2010–2012	3.3 (2–4.8)	Zimmer
Odgaard et al. 2018	2007–2014	2	Avon
Metcalfe et al. 2018	1996–2014	2–18	Avon
Middleton et al. 2018	2003–2014	5.6 (2.9–14.2)	Avon
**Registries**			
England/Wales (Baker et al. 2012)	2003–2010		Multiple
AOS (Australian Orthopaedic Association 2017)	1999–2016		Multiple
New Zealand 2017 (Muir et al. 1999)	1999–2016		Multiple
